# Parental age effects on neonatal white matter development

**DOI:** 10.1016/j.nicl.2020.102283

**Published:** 2020-05-26

**Authors:** Oliver Gale-Grant, Daan Christiaens, Lucilio Cordero-Grande, Andrew Chew, Shona Falconer, Antonios Makropoulos, Nicholas Harper, Anthony N Price, Jana Hutter, Emer Hughes, Suresh Victor, Serena J Counsell, Daniel Rueckert, Joseph V Hajnal, A David Edwards, Jonathan O'Muircheartaigh, Dafnis Batalle

**Affiliations:** a Centre for the Developing Brain, School of Imaging Sciences & Biomedical Engineering, King's College London, United Kingdom; b MRC Centre for Neurodevelopmental Disorders, King’s College London, United Kingdom; c Department of Computing, Imperial College London, United Kingdom; d Department of Forensic and Neurodevelopmental Science, Institute of Psychiatry, Psychology & Neuroscience, King’s College London, United Kingdom

**Keywords:** Paternal age, Brain development, Diffusion MRI, Newborn, Bayley, White matter

## Abstract

**Objective:**

Advanced paternal age is associated with poor offspring developmental outcome. Though an increase in paternal age-related germline mutations may affect offspring white matter development, outcome differences could also be due to psychosocial factors. Here we investigate possible cerebral changes prior to strong environmental influences using brain MRI in a cohort of healthy term-born neonates.

**Methods:**

We used structural and diffusion MRI images acquired soon after birth from a cohort (n = 275) of healthy term-born neonates. Images were analysed using a customised tract based spatial statistics (TBSS) processing pipeline. Neurodevelopmental assessment using the Bayley-III scales was offered to all participants at age 18 months. For statistical analysis neonates were compared in two groups, representing the upper quartile (paternal age ≥38 years) and lower three quartiles. The same method was used to assess associations with maternal age.

**Results:**

In infants with older fathers (≥38 years), fractional anisotropy, a marker of white matter organisation, was significantly reduced in three early maturing anatomical locations (the corticospinal tract, the corpus callosum, and the optic radiation). Fractional anisotropy in these locations correlated positively with Bayley-III cognitive composite score at 18 months in the advanced paternal age group. A small but significant reduction in total brain volume was also observed in in the infants of older fathers. No significant associations were found between advanced maternal age and neonatal imaging.

**Conclusions:**

The epidemiological association between advanced paternal age and offspring outcome is extremely robust. We have for the first time demonstrated a neuroimaging phenotype of advanced paternal age before sustained parental interaction that correlates with later outcome.

## Highlights

• Advanced paternal age is associated with a range of later negative outcomes.
• It is not known if these negative outcomes are due to genetics or environment.
• We use neonatal diffusion MRI to demonstrate paternal age effect on white matter.
• The babies of older fathers had reduced fractional anisotropy in multiple areas.
• These changes correlated with cognitive outcome at 18 months.

## Graphical abstract

**Figure:**
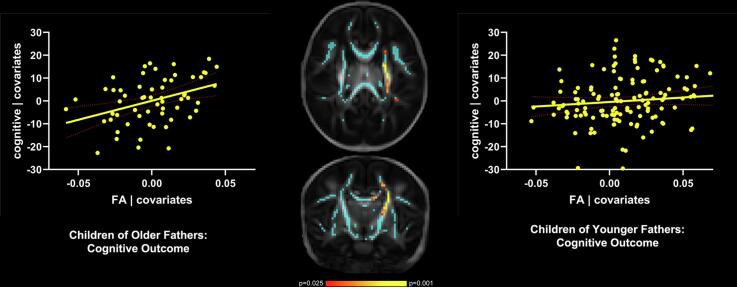

## 1 Introduction

Mean paternal age is increasing in many countries and advanced paternal age is strongly associated with an increased risk to offspring of multiple neurodevelopmental disorders including schizophrenia (Fountoulakis et al., 2018), bipolar disorder (Chudal et al., 2014) and autism (Khandwala et al., 2018), as well as non-clinical negative outcomes such as poor academic achievement (D'Onofrio et al., 2014).

Despite ample epidemiological evidence, the mechanism by which this risk is conferred is not clear. There are possible genetic explanations; de novo mutations in humans at birth are predominantly of paternal origin, and the paternal rate of transmitting mutations increases with age (Kong et al., 2012). Mechanisms of transcriptional control, including DNA methylation, also become more error prone with age (Horvath, 2013). Preclinical models give weight to this theory – the offspring of paternally aged mice display a range of undesirable behavioural phenotypes, including anxiety (Foldi et al., 2019), impaired sociability (Janecka et al., 2015), and reduced novelty preference (Smith et al., 2009).

Comparatively few studies have observed associations of advanced maternal age and childhood neuropsychiatric outcome (Sandin et al., 2012). When observed, some authors have attributed this association to the confounding effects of paternal age and preterm birth, both of which correlate with maternal age and with later neurodevelopmental disorder (Hultman et al., 2011).

Although the existence of a relationship between parental age and outcome appears to be certain, the nature of this relationship is unclear. The vast majority of authors find a non-linear relationship, with a certain cutoff age at which risk increases. This cutoff age varies considerably from study to study however (de Kluiver et al., 2017).

There is existing evidence of an influence of paternal age on neuroimaging phenotype. Shaw et al. (2012) reported associations of both paternal and maternal age with structural imaging in a cohort of 171 typically developing adolescents, reporting an “inverted U” shape association between parental age and grey matter volume, as well as negative associations between advanced paternal age and cortical surface area, and advanced maternal age and cortical thickness (Shaw et al., 2012). Recently, Kojima and colleagues used a cohort of 78 adult males with and without autism and demonstrated an association of advanced paternal age with reduced cortical thickness in the posterior cingulate cortex of the group with autism (Kojima et al., 2019), and Yassin and colleagues presented an analysis of the association of paternal age with diffusion imaging parameters in the same cohort and found a positive linear association between paternal age (but not maternal age), radial diffusivity and mean diffusivity observed in individuals with autism (Yassin et al., 2019).

Neonatal MRI is an attractive technique for dissecting the influences of heritable and environmental factors as at the time of image acquisition the majority of psychosocial influences are yet to occur. Fractional anisotropy (FA) changes in the neonatal period, representative of altered white matter microstructure, correlate with a large number of later outcomes, including intellectual ability and risk of psychiatric disease (Fusar-Poli et al., 2011). One group has investigated the influence of paternal and maternal age on brain volume soon after birth and found no association (Knickmeyer et al., 2017), however the effect of advanced paternal or maternal age on neonatal white matter microstructure has to our knowledge not previously been investigated.

In this study we used structural and diffusion MRI to investigate the association of advanced paternal age (the upper quartile of our cohort) with diffusion MRI characteristics in a cohort studied as part of the Developing Human Connectome Project (dHCP) (Hughes et al., 2017) using tract based spatial statistics (TBSS).

## 2 Methods

### 2.1 Sample

This study is based on a prospective sample of neonates participating in the Developing Human Connectome Project (dHCP, http://www.developingconnectome.org/). This project has received ethical approval (14/LO/1169, IRAS 138070), and written informed consent was obtained from parents.

533 individuals were included in the project at the time of this study. 105 individuals were excluded due to being born preterm (<37 weeks gestation) or due to complications during pregnancy or birth, and of the 428 individuals remaining, a complete set of imaging and demographic data including paternal and maternal age at birth was available for 275 individuals, who were included in the study. There were no significant differences in sex, ethnicity or age at scan between individuals included and excluded. Individuals were analysed in two groups based on the upper quartile and lower 3 quartiles of parental age at birth; for paternal age ≥38 years (n = 89) and <38 years (n = 186) and for maternal age ≥37 years (n = 82) and <37 years (n = 193).

### 2.2 Image acquisition

MRI data from each neonate was acquired on a Phillips 3-Tesla Achieva system (Philips Medical Systems, Best, The Netherlands) at the Evelina Newborn Imaging Centre, Evelina London Children’s Hospital. All infants were scanned during natural sleep without sedation using a dedicated protocol as previously described by our group (Hughes et al., 2017), including a bespoke transport system, positioning device and a customized 32-channel receive coil with a custom-made acoustic hood. All scans were supervised by a neonatal nurse and/or paediatrician who monitored heart rate, oxygen saturation and temperature throughout the scan.

T2-weighted images were obtained using a Turbo Spin Echo (TSE) sequence, acquired in two stacks of 2D slices (in sagittal and axial planes), using parameters: TR = 12 s, TE = 156 ms, SENSE factor 2.11 (axial) and 2.58 (sagittal) with overlapping slices (resolution 0.8 × 0.8 × 1.6 mm^3^), and diffusion images were acquired using parameters TR = 3800 ms, TE = 90 ms, SENSE factor = 1.2, multiband factor = 4, resolution 1.5 × 1.5 × 3.0 mm^3^ with 1.5 mm slice overlap. Diffusion gradient encoding included images collected at b = 0 s/mm^2^ (20 repeats), b = 400 s/mm^2^ (64 directions), b = 1000 s/mm^2^ (88 directions), b = 2600 s/mm^2^ (128 directions), and images were reconstructed to a final resolution of 1.5 × 1.5 × 1.5 mm^3^ (Hutter et al., 2018).

### 2.3 Image processing

Structural images were analysed using an automated processing pipeline optimised for neonates as previously described (Makropoulos et al., 2018). In brief, motion and bias corrected T2w images were brain extracted, and then segmented into 9 tissue types (cerebrospinal fluid, cortical grey matter, white matter, background, ventricle, cerebellum, deep grey matter, brainstem and amygdala & hippocampus) using the Draw-EM algorithm, an open source software optimised for neonatal brain segmentation (Makropoulos et al., 2014). The total brain volume and volumes of the grey matter, white matter, cerebellum, basal ganglia and ventricles (“regional volumes” hitherto) were then used for statistical analysis.

Diffusion MRI was pre-processed as previously described (Kelly et al., 2019). Briefly, images were denoised (Veraart et al., 2016), Gibbs ringing suppressed (Kellner et al., 2016) and reconstructed using a slice-to-volume motion correction technique that uses a bespoke spherical harmonics and radial decomposition (SHARD) method, together with outlier rejection, distortion and slice profile correction (Christiaens et al., 2019). Diffusion tensors were reconstructed and non-linearly registered to a population-based template using DTI-TK (Zhang et al., 2006). Mean diffusivity (MD), axial diffusivity (AD), radial diffusivity (RD) and FA maps for each subject were subsequently generated in template space, and re-integrated into the FSL TBSS pipeline optimised for the neonatal brain (Ball et al., 2010).

### 2.4 Followup

Neurodevelopmental assessment was offered to all participants at 18 months. Neurodevelopmental performance was assessed using the Bayley Scales of Infant and Toddler Development, Third Edition (Bayley, 2006). The cognitive, motor and language composite scores were included as outcome measures in this study. Index of multiple deprivation (IMD), (a geographically defined composite social risk score comprising data on income, employment, health, education, living environment and crime) was included as a cofactor in the regression model, using the mother’s address at the time of birth to calculate this (Abel et al., 2016).

### 2.5 Statistical analysis

Association of total and segmented regional brain volumes (white matter, grey matter, cerebellum, basal ganglia and cerebellum) with advanced parental age was performed using general linear model (GLM) in STATA 15. Gestational age at birth and post menstrual age at scan were always included as covariates. Maternal age was strongly co-linear with paternal age (r^2^ = 0.29); in order to dissect the specific influence of paternal age, maternal age was included as an additional covariate in tests of paternal age. Total brain volume was included as an additional covariate in correlations of regional brain volume with parental age.

TBSS analysis was performed using the *randomise* tool for nonparametric permutation inference in FSL, with 10,000 permutations per test. Threshold free cluster enhancement (optimised for 3D data) and family wise error (FWE) rate was applied to correct for multiple comparisons. All matrices contained gestational age at birth and post menstrual age at scan as covariates, and tests of paternal age were additionally corrected for maternal age. Sex had no effect on voxel-wise white matter microstructure in this cohort, so was not included in any matrix, nor was it associated with advanced paternal age (OR 0.87, 95% CI 0.52 – 1.44, p = 0.593). All p-values reported were FWE corrected. Cluster based inference was performed using FSL, with an initial threshold set at p < 0.05. Additional post-hoc statistical analysis was performed using STATA 15 and GraphPad Prism 8.

### 2.6 Data availability

The dHCP is an open-access project. The imaging and collateral data used in this study were included in the 2019 (second) dHCP data release, which can be downloaded by registering at https://data.developingconnectome.org/

## 3 Results

Individuals were split into two groups based on parental age at birth; for paternal age ≥38 years (n = 89) and <38 years (n = 186) and for maternal age ≥37 years (n = 82) and <37 years (n = 193). The demographic details of the cohort are shown in Table 1, and a frequency distribution of paternal ages is shown in Supplementary Fig. 1.

**Table 1:** Demographic details of participants.

	n
Male	145
Female	130

	Median (IQR)

Paternal age at birth (years)	36 (32–38)
Maternal age at birth (years)	34 (31–37)

Gestation at birth (weeks)	40.1 (39.0–40.6)
Age at scan (weeks)	40.9 (39.7–42.0)

**Fig. 1:**
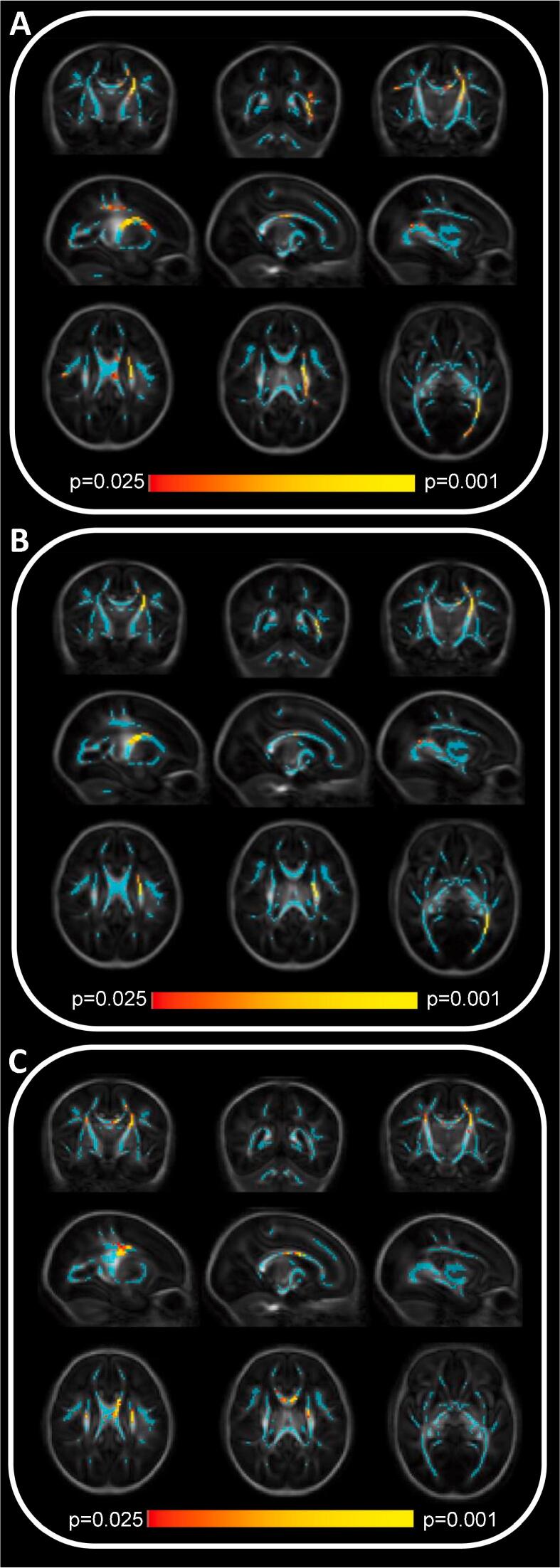
Association between paternal age and (A) fractional anisotropy (FA), (B) mean diffusivity (MD), and (C) radial diffusivity. Colours represent areas of white matter tracts where FA is reduced, MD increased or RD increased in the offspring of older fathers. Areas of significant difference on the white matter skeleton (blue) are shown, with p values indicated by the colour bar. Top to bottom – coronal (anterior to posterior), sagittal (L to R), and axial (superior to inferior) images. There were no significant areas of difference in axial diffusivity (not shown). (For interpretation of the references to colour in this figure legend, the reader is referred to the web version of this article.)

TBSS revealed 3 clusters of significant difference in FA between the offspring of older and younger fathers after accounting for maternal age, age at birth and age at scan as confounding factors, with lower FA values observed in the offspring of older fathers: within the genu of the corpus callosum; the left corticospinal tract; and the left optic radiation (Fig. 1A). Similar patterns of difference were observed in MD and RD (with higher values observed in the offspring of older fathers) however no areas of statistical significance were observed in AD (Fig. 1B, C).

Applying the same methodology to maternal age with paternal age as a covariate, did not reveal any significant difference between groups. There was no linear association between paternal or maternal age and any of the measured diffusion imaging metrics (FA, MD, RD, AD).

**Fig. 2:**
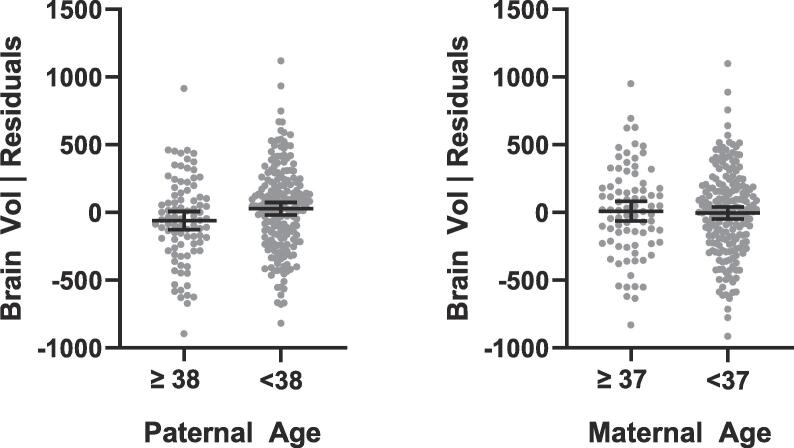
Residuals of brain volume (mm^3^) of individuals with (A) paternal age at birth ≥38 and <38 years and (B) maternal age at birth ≥37 and <37 years. Volume corrected for age at birth, age at scan, and maternal age (for test of paternal age) or paternal age (for test of maternal age).

To test possible influences of advanced paternal age on brain macrostructure, the associations between advanced parental age and total and relative regional brain volumes (white matter, grey matter, cerebellum, basal ganglia and cerebellum) were checked. There was a small but significant difference in total brain volume observed between individuals of older and younger paternal age (r^2^ = 0.02, p = 0.013, Fig. 2), with the offspring of older fathers having a slightly reduced total brain volume. There was however no significant difference in relative regional brain volumes, and no significant difference in total brain volume or regional volumes between individuals of older and younger maternal age (Supplementary Table 1).

**Fig. 3:**
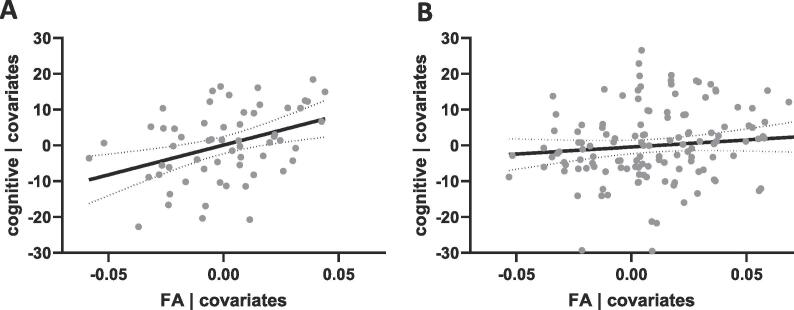
Association between corticospinal tract cluster FA and 18 month cognitive outcome for individuals with (A) PA ≥38 (r^2^ = 0.15, p = 0.002) and (B) PA <38 (r^2^ < 0.01, p = 0.224). Residual FA values are shown adjusted for age at scan, age at birth and maternal age. Cognitive outcome shown adjusted for Index of Multiple Deprivation score. 95% CI of linear regression indicated by dotted lines.

Outcome data was available for 196 individuals (43 were <18 months at the time of analysis, 36 lost to or declined followup). There was no significant difference between group mean Bayley-III cognitive scores – paternal age ≥38 mean 100.6 (SD 10.1), paternal age <38 mean 101.05 (SD 10.2). However, there was a significant between group interaction between cognitive scores and average FA in the TBSS cluster observed in the corticospinal tract (r^2^ = 0.08, p = 0.013) with a positive linear association between FA and cognitive outcome in the group with paternal age ≥38 (r^2^ = 0.15, p = 0.002, Fig. 3). There was no association identified between diffusion imaging metrics and Bayley-III language or motor scores.

## 4 Discussion

Healthy term born infants show differences in cerebral microstructure and macrostructure associated with advanced paternal age, before sustained paternal social and family interactions have occurred.

Maximal clusters of white matter microstructural differences were observed in the corticospinal tract, the genu of the corpus callosum and the optic radiation. The significance of these locations relates to their status as early developing areas (Branson, 2013) rather than to their function in later life. During the first few postnatal months white matter growth is complex, with dendritic growth from cell bodies and arborisation occurring simultaneously with rapid proliferation of glial cells. These parallel processes result in white matter density (which correlates predominantly with MD) and organisation (which correlates predominantly with FA) changing at differing rates (Batalle et al., 2019), which may explain the slightly different patterns of FA and MD association with paternal age demonstrated here. It is notable that whilst multiple areas of significant difference in RD were identified no difference in AD was observed. RD is thought to relate more to myelin integrity than AD (Janve et al., 2013, Song et al., 2003, Winklewski et al., 2018). At the time of scan acquisition myelination is beginning to occur rapidly, causing subtle inter individual differences to manifest. Changes in the trajectory of early development measured by RD have been specifically associated with cognitive outcome in early life (Girault et al., 2019).

The finding of a small but significant reduction in total brain volume in the advanced paternal age group is further evidence of an alteration in brain maturation.

Despite the presence of a paternal age effect in this cohort, there is no maternal age effect observed. This is similar to the findings of Yassin and colleagues in a cohort of adult males with autism (Yassin et al., 2019). A possible hypothesis is that the different replication schedules of sperm and oocytes are mediating different effects on offspring white matter. At age 20, spermatic chromosomes (which do not have DNA repair mechanisms) have undergone 150 replications, compared to 610 by age 40 (Kong et al., 2012). Oocytes however undergo 22 mitotic replications regardless of their age at fertilisation (Vogel, 1997).

Advanced maternal age is associated with an increased incidence of perinatal risk factors such as premature birth and intrauterine growth restriction (Aldous and Edmonson, 1993) which are in turn associated with altered brain development in early life (Batalle et al., 2017). Our study by design excludes premature births and other perinatal risk factors to avoid this confounding effect.

We have observed two markedly different associations between measures of diffusivity in an early-myelinating tract and cognitive function at 18 months in the groups of paternal age ≥ or <38 years. This aligns with the “cumulative risk” hypothesis of neurodevelopmental disease, which proposes that once sufficient environmental risk factors are experienced (of which advanced paternal age is one) an individual is in an *at risk* state from which an adverse phenotype can emerge (Bolte et al., 2019). This may explain how subtle differences, such as those observed here, contribute to significantly different outcomes. A similar pattern of association between neonatal white matter microstructure and later cognitive outcome (seen only in at-risk individuals) has also been observed in preterm babies (Keunen et al., 2017).

Paternal age related de novo mutations probably confer only a modest increased risk for developmental disorder (Taylor et al., 2019). It is likely that there are also psychosocial explanations for the considerable differences in offspring outcome observed with increased paternal age (Gratten et al., 2016). Older parents are more likely to die before their children reach adulthood (Schmidt et al., 2011), and intra-familial relationships are more discordant in families with high generational age gap (Boivin et al., 2009). Humans do not reproduce at random, and there is evidence that men who delay fatherhood may do so due to themselves experiencing adversity during early adulthood, or struggling to find a mate (Puleo et al., 2008). Fathers in this group therefore may not be homogenous with younger fathers in terms of their own genetic risk.

White matter FA has a strong genetic basis, and changes similar to those observed here have been correlated with combined genetic risk scores in both adults and infants (Gilmore et al., 2018, Kochunov et al., 2015). Studies such as this one, using neuroimaging phenotype prior to the majority of environmental influence, allow for an intermediate phenotype between genetic risk and neurodevelopmental outcome to be measured, and are a promising avenue for future study.

In this study we have observed a group effect of advanced paternal age. This is in keeping with the majority of literature, however there is little agreement as to what age is considered “advanced”. For convenience many researchers analyse fathers in groups of 10 years, so 40 or 50 are commonly proposed cutoff ages (Oldereid et al., 2018, Urhoj et al., 2017), but authors have however proposed a far wider range. Taking the association between paternal age and autism spectrum disorder as an example, 35 (Lampi et al., 2013), 39 (Grether et al., 2009), 40 (Puleo et al., 2012), 50 (Frans et al., 2013, Hultman et al., 2011), and 55 years (Idring et al., 2014) have all been proposed from good quality studies using population registers. The methodology of these studies also differs significantly, with many comparing fathers over a chosen age with fathers of an “ideal” age, the latter usually arbitrarily defined as 20–29 (Idring et al., 2014). Rather than favour any previously proposed age we therefore decided to divide our cohort by the upper quartile of paternal age.

Our study is methodologically robust – we used a large sample size with high follow-up rates, and a state-of-the-art protocol acquired on the same MRI scanner for each subject (Hutter et al., 2018). We have also applied the more stringent 3D optimisation of threshold free cluster enhancement throughout our analysis (Smith and Nichols, 2009) – voxelwise significance images using 2D optimisation are shown in Supplementary Fig. 2.

Previous evidence of the relationship between paternal age and childhood adverse outcome is primarily from large scale epidemiology studies, and this link has been replicated in multiple cohorts in different countries (Janecka et al., 2017). These data show an influence of advanced paternal age on the brain before the age of substantial paternal social interactions, with an associated effect on cognitive outcome at 18 months. This may offer a path to improving our understanding of the important link between paternal age and offspring neurodevelopment.

## Funding

This work was supported by the 10.13039/501100000781European Research Council under the European Union's Seventh Framework Programme (FP7/20072013)/ERC grant agreement no. 319456 (dHCP project). The authors acknowledge infrastructure support from the 10.13039/501100000272National Institute for Health Research (NIHR) Mental Health Biomedical Research Centre (BRC) at South London, 10.13039/100009362Maudsley NHS Foundation Trust, King's College London and the NIHR-BRC at Guys and St Thomas’ Hospitals NHS Foundation Trust (GSTFT). The authors also acknowledge support in part from the Wellcome Engineering and Physical Sciences Research Council (EPSRC) Centre for Medical Engineering at Kings College London [WT 203148/Z/16/Z], MRC strategic grant [MR/K006355/1], Medical Research Council Centre grant [MR/N026063/1], the Department of Health through an NIHR Comprehensive Biomedical Research Centre Award (to Guy’s and St. Thomas’ National Health Service (NHS) Foundation Trust in partnership with King’s College London and King’s College Hospital NHS Foundation Trust), the Sackler Institute for Translational Neurodevelopment at King’s College London and the European Autism Interventions (EU-AIMS) trial and the EU AIMS-2-TRIALS, a European Innovative Medicines Initiative Joint Undertaking under Grant Agreements No. 115300 and 777394, the resources of which are composed of financial contributions from the European Union’s Seventh Framework Programme (Grant FP7/2007–2013). OGG is supported by a grant from the UK Medical Research Council [MR/P502108/1]. JOM and DE received support from the Medical Research Council Centre for Neurodevelopmental Disorders, King’s College London [MR/N026063/1]. JOM is supported by a Sir Henry Dale Fellowship jointly funded by the Wellcome Trust and the Royal Society [206675/Z/17/Z]. DB received support from a Wellcome Trust Seed Award in Science [217316/Z/19/Z]. The views expressed are those of the authors and not necessarily those of the NHS, the National Institute for Health Research or the Department of Health. The funders had no role in the design and conduct of the study; collection, management, analysis, and interpretation of the data; preparation, review, or approval of the manuscript; and decision to submit the manuscript for publication.

## CRediT authorship contribution statement

**Oliver Gale-Grant:** Methodology, Writing - original draft, Visualization, Formal analysis. **Daan Christiaens:** Software, Resources, Writing - review & editing. **Lucilio Cordero-Grande:** Software, Resources. **Andrew Chew:** Investigation, Writing - review & editing. **Shona Falconer:** Investigation, Methodology. **Antonios Makropoulos:** Software, Validation. **Nicholas Harper:** Data curation, Methodology. **Anthony N Price:** Methodology. **Jana Hutter:** Methodology, Validation. **Emer Hughes:** Conceptualization, Methodology. **Suresh Victor:** Investigation, Writing - review & editing. **Serena J Counsell:** Conceptualization, Writing - review & editing. **Daniel Rueckert:** Conceptualization, Software. **Joseph V Hajnal:** Conceptualization, Methodology, Software, Resources. **A David Edwards:** Conceptualization, Resources, Supervision, Writing - review & editing. **Jonathan O’Muircheartaigh:** Methodology, Investigation, Supervision, Writing - review & editing. **Dafnis Batalle:** Methodology, Investigation, Supervision, Writing - review & editing.

## Declaration of Competing Interest

The authors declare that they have no known competing financial interests or personal relationships that could have appeared to influence the work reported in this paper.
