# Supplementary figures and images for: Parental age effects on neonatal white matter development
Source: Neuroimage Clin. 2020 May 26;27:102283. doi: 10.1016/j.nicl.2020.102283 (PMC7284122; doi:10.1016/j.nicl.2020.102283)

## Slide 1
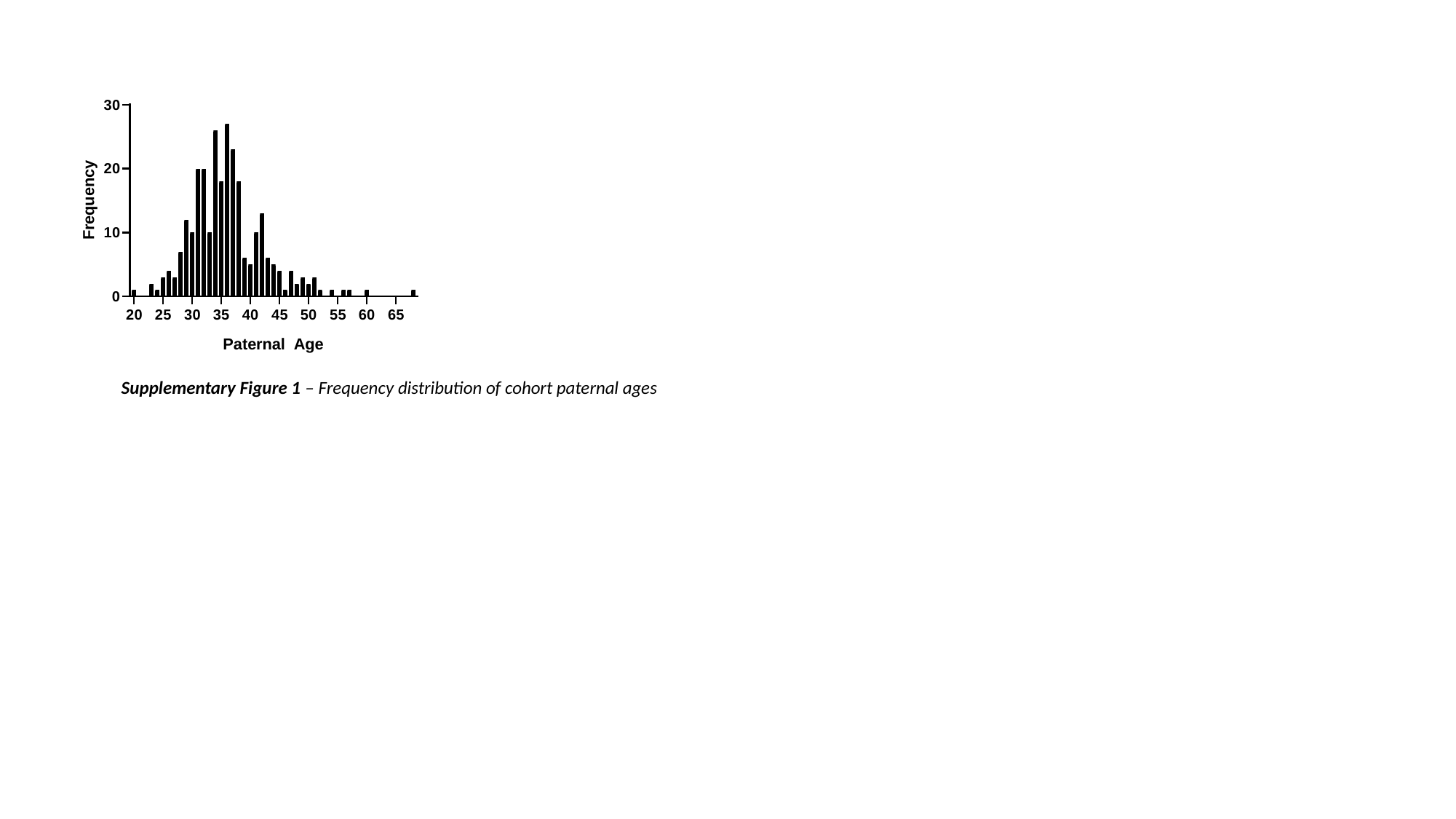

Supplementary Figure 1 – Frequency distribution of cohort paternal ages

Supplement: Supplementary data 1 [file mmc1.pptx]
