# Supplementary material for: Parental age effects on neonatal white matter development
Source: Neuroimage Clin. 2020 May 26;27:102283. doi: 10.1016/j.nicl.2020.102283 (PMC7284122; doi:10.1016/j.nicl.2020.102283)
Supplement: Supplementary data 2 [file mmc2.pptx]

## Slide 1
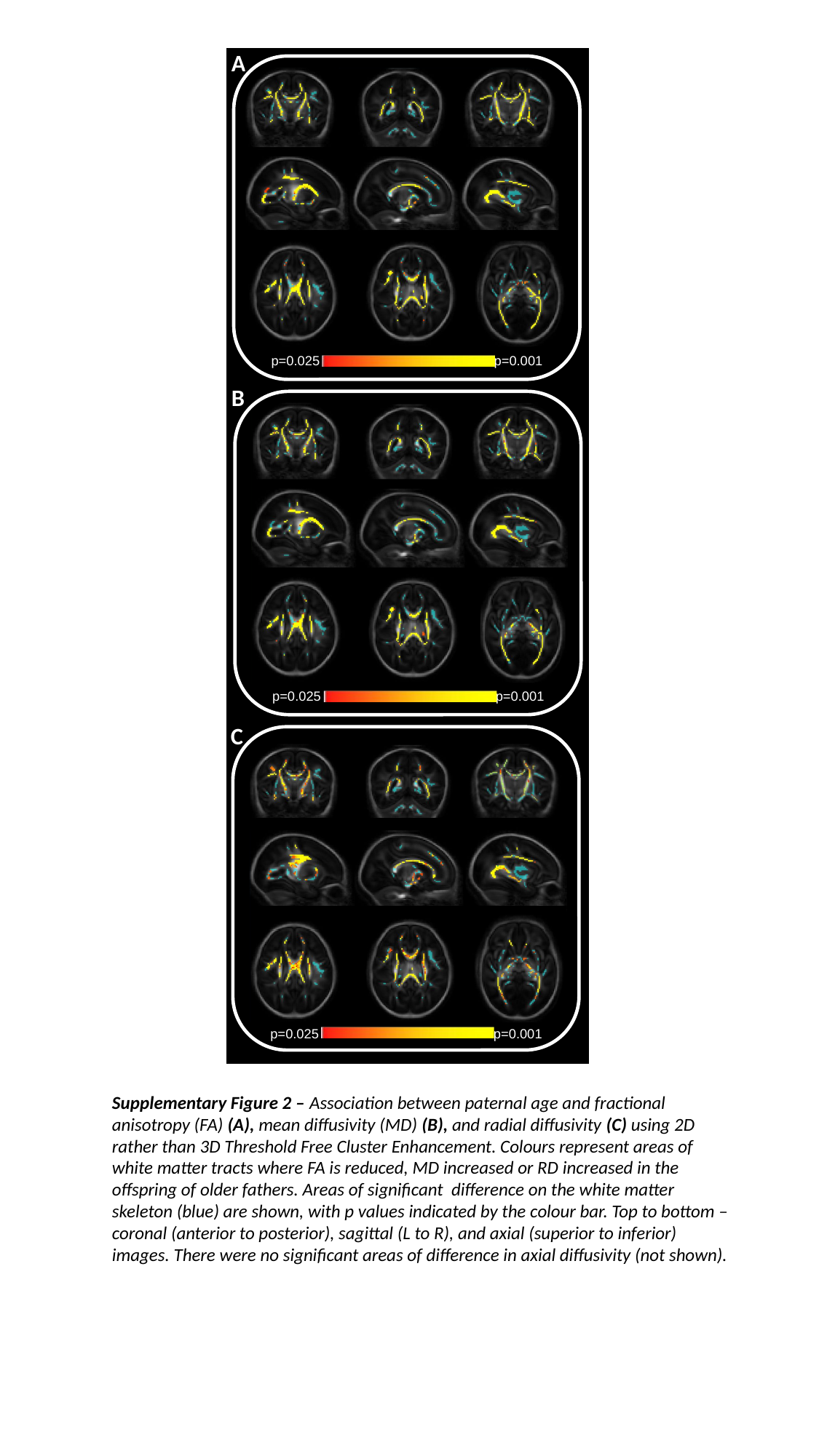

A
p=0.025 p=0.001
B
p=0.025 p=0.001
C
p=0.025 p=0.001
Supplementary Figure 2 – Association between paternal age and fractional anisotropy (FA) (A), mean diffusivity (MD) (B), and radial diffusivity (C) using 2D rather than 3D Threshold Free Cluster Enhancement. Colours represent areas of white matter tracts where FA is reduced, MD increased or RD increased in the offspring of older fathers. Areas of significant difference on the white matter skeleton (blue) are shown, with p values indicated by the colour bar. Top to bottom – coronal (anterior to posterior), sagittal (L to R), and axial (superior to inferior) images. There were no significant areas of difference in axial diffusivity (not shown).
