# Supplementary material for: Parental age effects on neonatal white matter development
Source: Neuroimage Clin. 2020 May 26;27:102283. doi: 10.1016/j.nicl.2020.102283 (PMC7284122; doi:10.1016/j.nicl.2020.102283)
Supplement: Supplementary data 3 [file mmc3.pdf]

Supplementary Table 1 *– Associations of paternal age (A) and maternal age (B) with regional (white matter, grey matter, cerebellum, basal ganglia and cerebellum) and total brain volumes (mm^3) caculated by general linear model. Paternal age and maternal age are considered as binary variables (paternal age < or ≥ 38 years, maternal age < or ≥ 37 years). Age at scan and age at birth were included in all models. Tests of paternal age were additionally corrected for maternal age. Tests of regional brain volumes included total brain volume as a covariate.*

A

	**Coef**	**95% CI**	**p**
**Whole Brain**	-12022.90	-21468.3 - -2577.527	**0.013**
**Grey Matter**	17.59	-1163.23 - 1198.42	0.977
**White Matter**	129.46	-962.56 - 1221.48	0.816
**Cerebellum**	59.64	-451.29 - 570.57	0.819
**Basal Ganglia**	-335.25	-677.65 - 7.15	0.055
**Ventricles**	118.38	-497.75 - 734.5	0.706

B

	**Coef**	**95% CI**	**p**
**Whole Brain**	817.87	-7647.14 – 9282.90	0.850
**Grey Matter**	-436.19	-4282.95 – 3410.56	0.824
**White Matter**	723.35	-2934.43 – 4381.05	0.698
**Cerebellum**	267.83	-312.18 – 847.85	0.365
**Basal Ganglia**	-103.28	-671.49 – 464.94	0.722
**Ventricles**	243.71	-334.75 – 822.19	0.409
